# Dynamic Feature Fusion for Sparse Radar Detection: Motion-Centric BEV Learning with Adaptive Task Balancing

**DOI:** 10.3390/s26030968

**Published:** 2026-02-02

**Authors:** Yixun Sang, Junjie Cui, Yaoguang Sun, Fan Zhang, Yanting Li, Guoqiang Shi

**Affiliations:** School of Mechanical and Electrical Engineering, North University of China, Taiyuan 030051, China; 15035660768@163.com (Y.S.); 17200585117@163.com (Y.S.); sz202301105@st.nuc.edu.cn (F.Z.); s202401034@st.nuc.edu.cn (Y.L.); s202301028@st.nuc.edu.cn (G.S.)

**Keywords:** 4D radar perception, autonomous driving, motion-aware learning

## Abstract

This paper proposes a novel motion-aware framework to address key challenges in 4D millimeter-wave radar detection for autonomous driving. While existing methods struggle with sparse point clouds and dynamic object characterization, our approach introduces three key innovations: (1) A Bird’s Eye View (BEV) fusion network incorporating velocity vector decomposition and dynamic gating mechanisms, effectively encoding motion patterns through independent XY-component convolutions; (2) a gradient-aware multi-task balancing scheme using learnable uncertainty parameters and dynamic weight normalization, resolving optimization conflicts between classification and regression tasks; and (3) a two-phase progressive training strategy combining multi-frame pre-training with sparse single-frame refinement. Evaluated on the TJ4D benchmark, our method achieves 33.25% mean Average Precision (mAP)_3D_ with minimal parameter overhead (1.73 M), showing particular superiority in pedestrian detection (+4.16% AP) while maintaining real-time performance (24.4 FPS on embedded platforms). Comprehensive ablation studies validate each component’s contribution, with thermal map visualization demonstrating effective motion pattern learning. This work advances robust perception under challenging conditions through principled motion modeling and efficient architecture design.

## 1. Introduction

Autonomous vehicles require real-time perception of their surrounding environment through a multi-modal sensor system, where object detection technology serves as a core component, tasked with accurately identifying and localizing road participants (e.g., vehicles, pedestrians, traffic signs) from vast amounts of sensor data. Traditional vision-based detection solutions relying on 2D cameras and convolutional neural networks (CNNs) achieve high detection accuracy under normal lighting conditions. However, their inherent limitations become increasingly apparent in complex scenarios: monocular cameras lack depth perception, leading to significant errors in target distance estimation. To address similar challenges in perception tasks, recent advances in deep learning have shown that generating depth information from regular RGB images—either via true RGB-D sensors or pseudo RGB-D techniques—can significantly improve recognition and diagnostic performance in facial analysis tasks, demonstrating the value of multi-modal depth cues in enhancing 2D image understanding [1,2]. While stereo cameras can compute depth information through disparity, their baseline length constraints cause quadratic growth in ranging errors at high speeds (>80 km/h). More critically, in low-light night-time environments or extreme lighting conditions—such as the abrupt brightness changes (up to $10^{4}$ lux) at tunnel entrances/exits or lens flare caused by direct sunlight at noon, which reduces the image signal-to-noise ratio (SNR) by over 15 dB—the limited dynamic range of typical automotive cameras (around 70 dB) falls short of real-world road scenarios (often exceeding 120 dB). This results in overexposed or underexposed images, significantly degrading feature extraction quality and even causing false or missed detections.

To compensate for the shortcomings of visual perception, LiDAR (Light Detection and Ranging) leverages its active laser emission capability to generate high-precision 3D point cloud data (single-frame point cloud density up to 260 k points), demonstrating unique advantages in depth perception and obstacle contour modeling. A 64-beam LiDAR can achieve centimeter-level ranging accuracy, and when combined with point cloud segmentation algorithms, it effectively parses complex scene structures. However, solid-state LiDARs (e.g., Livox Horizon) perform poorly in adverse weather conditions such as rain or snow: raindrops (rainfall > 25 mm/h) cause beam scattering, while snowflakes generate false point clouds, posing significant safety risks for emergency braking in high-speed scenarios. Millimeter-wave radar, with its strong electromagnetic wave penetration and all-weather operation capabilities, leverages the Doppler effect (velocity measurement accuracy of ±0.1 m/s) to precisely measure relative target speeds. Developing detection methods for millimeter-wave radar is crucial for enhancing system redundancy.

In recent years, significant progress has been made in LiDAR-based object detection for autonomous driving, with pillar-based feature representation methods gaining widespread adoption due to their efficient grid processing capabilities (processing speed improved by 3×). Traditional methods primarily rely on spatial geometric features (e.g., coordinates, reflection intensity) and statistical properties (e.g., density, variance) of point clouds. However, they still face two critical challenges in complex dynamic scenarios: (1) confusion between static and dynamic targets leading to higher false alarm rates, and (2) insufficient instantaneous state perception of moving objects limiting trajectory prediction accuracy.

Beyond sensor perception, predicting pedestrian behavior is crucial for autonomous navigation. Recent deep learning models, such as Social NSTransformers [3], the Social Entropy Informer [4], and the Social Informer [5], capture social interactions and stochasticity in pedestrian motion, improving trajectory prediction accuracy and robustness. These advances underscore the value of combining predictive models with multi-modal sensing for enhanced situational awareness. However, if one sensor fails, performance is compromised.

Four-dimensional millimeter-wave radar is an advanced sensor technology that builds upon traditional millimeter-wave radar (capable of detecting distance, velocity, and horizontal azimuth) by adding vertical height perception (pitch angle resolution of 1.5°), forming a four-dimensional detection system (range, velocity, horizontal angle, vertical angle). By measuring vertical angles, it reduces misjudgments; it operates stably in all weather conditions, unaffected by lighting, rain, snow, or fog; its effective detection range exceeds 300 m (up to 350 m for trucks), enabling early perception of distant vehicles or obstacles. However, millimeter-wave radar point clouds are extremely sparse (approximately 200 points per frame) and noisy (SNR < 10 dB).

**Figure 1:**
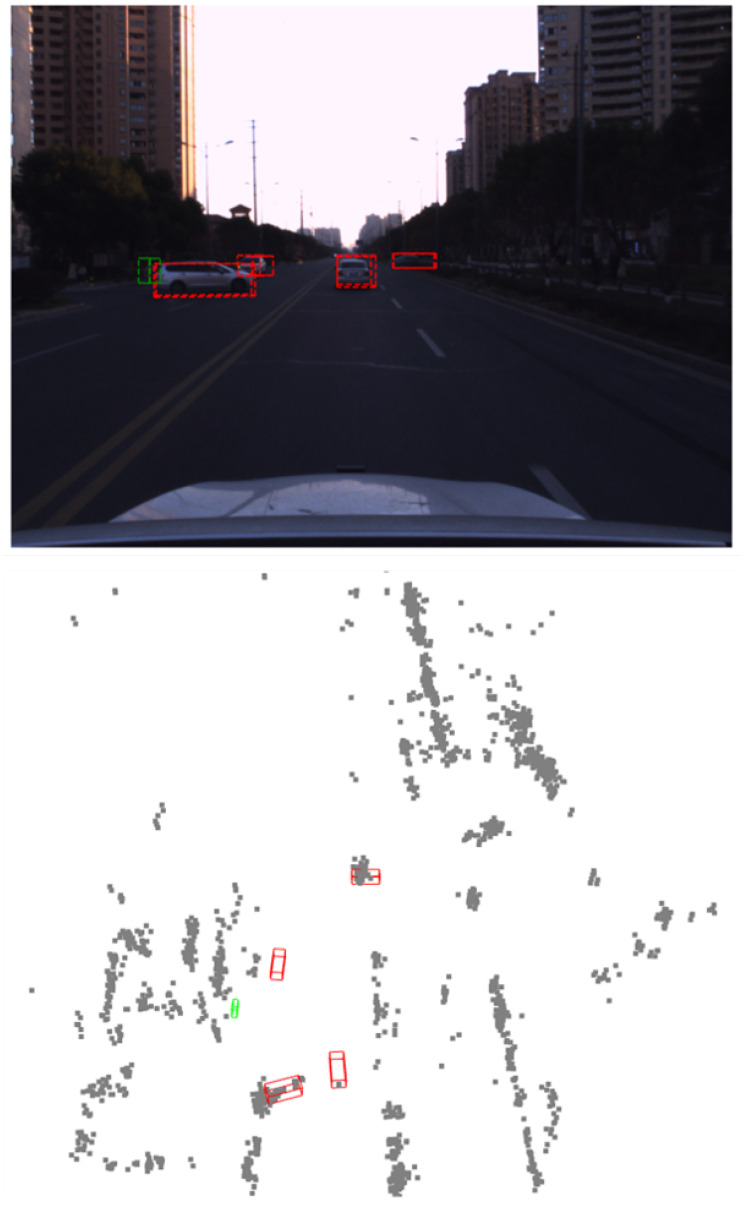
Visualization of detection results. The upper image shows the detection results projected onto the camera image, while the lower image displays the detection results on noisy millimeter-wave radar point clouds. Red boxes represent vehicle detections, and green boxes represent pedestrian detections.

Nevertheless, existing millimeter-wave radar detection methods suffer from three key limitations: (1) underutilization of velocity information, which remains at the level of simple feature concatenation, leading to inadequate spatial distribution modeling of velocity vectors and confusion in dynamic target features; (2) rigid weight allocation strategies in multi-task loss functions, causing gradient conflicts; and (3) insufficient representation learning for foreground targets due to the extreme sparsity of radar point clouds. As shown in Figure 1, our method achieves accurate detection on sparse radar point clouds.

This paper proposes a motion-aware spatiotemporal joint feature learning framework, achieving breakthrough improvements in millimeter-wave radar detection performance through a deep coupling mechanism between velocity fields and spatial features. The main innovations include the following:

- **Motion-enhanced BEV encoding network**: A velocity vector decomposition encoding strategy is proposed, where velocity vectors are decomposed into XY components for independent convolutional encoding, and motion feature maps are constructed through channel concatenation. A dynamic feature fusion gating mechanism is designed, using intensity features activated by sigmoid as gating signals to achieve dynamic weighted fusion of motion and density features.
- **Gradient-aware multi-task balancing mechanism**: An uncertainty-weighted loss function is constructed, introducing learnable variance parameters for each loss term to achieve adaptive weighting. A dynamic gradient normalization strategy is proposed, dynamically adjusting weight allocation by monitoring changes in task gradient norms to prevent specific tasks from dominating the training process.
- **Two-stage progressive training strategy**: In the first stage, motion patterns are learned from dense point clouds, followed by migration to sparse point cloud scenarios in the second stage.

**Figure 2:**
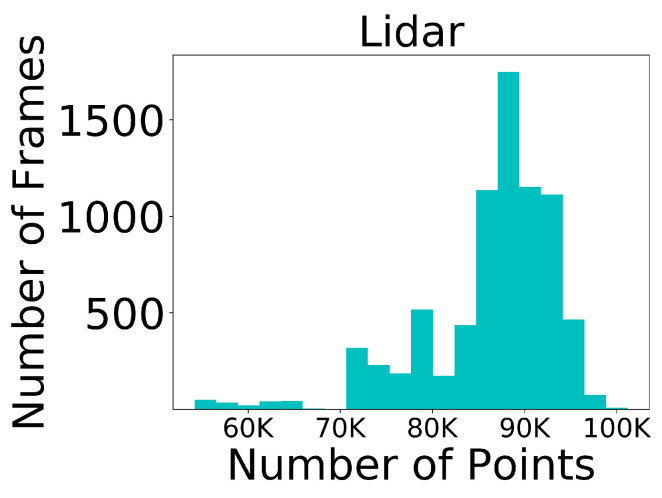
LiDAR point cloud statistics in the TJ4D dataset.

**Figure 3:**
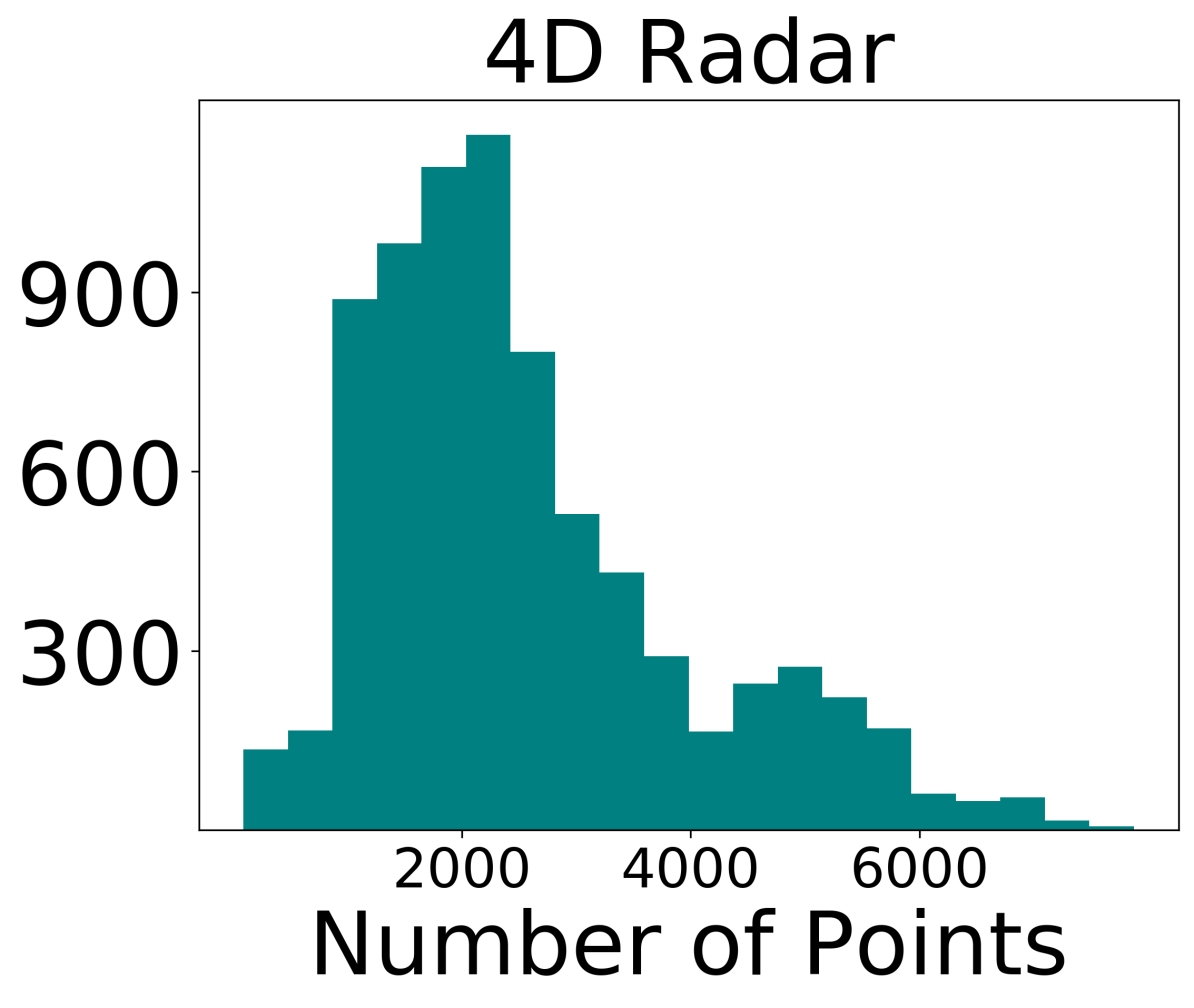
Millimeter-wave radar point cloud statistics in the TJ4D dataset.

**Evidence-to-claim alignment:** We explicitly link each contribution to empirical evidence to improve readability: the overall accuracy–efficiency trade-off is supported by Table 1, while the independent effectiveness of each proposed component is validated in Table 2. Dataset sparsity characteristics motivating our design choices are shown in Figure 2 and Figure 3.

**Discussion:** Figure 2 and Figure 3 highlight the substantial density gap between LiDAR and 4D radar in TJ4D: radar point clouds are far sparser and noisier, and moving-object evidence is often fragmented. This motivates (i) our motion-centric BEV encoding to make better use of Doppler cues for dynamic targets, and (ii) the multi-stage training strategy that first learns from temporally densified inputs and then adapts to single-frame inference.

## 2. Related Work

### 2.1. LiDAR Detection

LiDAR-based detection methods emphasize uncertainty modeling, temporal fusion, and multi-modal integration to improve robustness under adverse conditions. Given the structural similarity between millimeter-wave radar and LiDAR point clouds, many radar detection frameworks borrow design principles from LiDAR-based approaches, such as pillar-based encoding, attention mechanisms, and BEV representation learning. This explains why the LiDAR detection literature is often included in related work: it provides foundational techniques and architectural insights that inspire radar-specific adaptations. However, direct transplantation of LiDAR methods is insufficient because radar data exhibit unique characteristics such as Doppler velocity and severe sparsity, which require tailored solutions. Key trends include the following:

**Uncertainty-aware and semantic reasoning:** Feng et al. [15] proposed a spatial uncertainty model estimating annotation errors via generative methods. Mandalika et al. [16] introduced Bayesian graph networks with chain-of-thought reasoning for decision-making. Peng et al. [17] addressed inter-class confusion using probabilistic soft logic.

**Temporal and multi-frame fusion:** He et al. [18] presented a motion-guided sequential fusion method reducing redundancy through bidirectional aggregation. McCrae et al. [19] enhanced PointPillars with recurrent memory for dynamic object detection using multi-frame LiDAR sequences.

**Cross-modal fusion:** Bharadhwaj et al. [20] improved perception by fusing LiDAR and camera data with point cloud upsampling. Khan et al. [21] developed a real-time multi-modal detection system integrating visual and LiDAR features. Wu et al. [22] proposed virtual sparse convolution for efficient image-LiDAR fusion.

**Transformer-based sparse models:** Son et al. [23] introduced SparseVoxFormer, enabling efficient multi-modal fusion via sparse voxel Transformers. Zhang et al. [24] developed Uni3D for cross-domain generalization through semantic coupling-decoupling modules.

**Lightweight 3D Detection:** Chen et al. [25] proposed RailVoxelDet, demonstrating that carefully designed sparse voxelization and efficient backbones can achieve strong accuracy under constrained computational budgets, which is aligned with embedded real-time deployment requirements.

Current LiDAR detection approaches often overlook motion-aware encoding, relying heavily on static spatial features. Multi-task balancing strategies remain underdeveloped, and progressive training for sparse scenarios is rarely considered, limiting adaptability in long-range or degraded environments. LiDAR detection advances center on uncertainty-aware reasoning, temporal feature aggregation, and cross-modal fusion, with Transformer-based architectures emerging as a promising direction for sparse and heterogeneous data integration.

### 2.2. Radar Detection

Recent radar-based detection methods primarily address challenges of sparsity, noise, and motion-induced distortions through attention mechanisms, velocity-aware modeling, and cross-modality fusion. Representative approaches can be grouped as follows:

Additional recent works related to 4D radar detection/tracking and robustness include CenterRadarNet [26], RPFA-Net [27], VA-Net [28], and MSPFNet [29]. Dataset/benchmark efforts such as VoD [30] further support multi-class evaluation. We also note several recent preprints on multi-modal fusion, cross-domain generalization, and practical safety reasoning [31,32,33,34,35,36]. For completeness, we cite a related LiDAR-only study as well [37].

**Physics-guided and interpretable learning:** Beyond purely data-driven feature design, incorporating explicit physical constraints into learning objectives has been explored to improve interpretability and reliability in safety-critical diagnostics. For example, You et al. [38] embedded physically derived motion equations as loss constraints to guide representation learning. While this work targets bearing fault diagnosis, it motivates future radar perception research to integrate domain laws (when available) for more credible and interpretable modeling.

**Attention-based pillar encoding:** Li et al. [39] introduced PillarDAN, integrating dual attention mechanisms to enhance spatial-channel feature representation for radar point clouds. Musiat et al. [40] designed RadarPillars with hierarchical scaling strategies for efficient feature extraction. Bi et al. [41] proposed MAFF-Net (originally a LiDAR+Camera multi-modal fusion method adapted to radar contexts), leveraging Sparse Pillar Attention (SPA) and Cluster Query Cross-Attention (CQCA), though the attention overhead may limit embedded deployment efficiency.

**Velocity-aware and motion compensation:** Tan et al. [42] developed a multi-frame detection framework that improves point cloud density via velocity compensation and inter-frame matching. Wang et al. [43] proposed DADAN, employing dynamic augmentation and density-aware attention to distinguish foreground from background. Pan et al. [44] introduced RaTrack, a radar-based tracking method that bypasses 3D bounding boxes by focusing on motion segmentation and clustering, achieving superior performance on the View-of-Delft dataset.

**Cross-modality fusion and knowledge transfer:** Chae et al. [45] introduced 3D-LRF, fusing LiDAR and radar features with a weather-aware gating network. Xu et al. [46] presented SCKD, a semi-supervised distillation framework transferring LiDAR knowledge to radar models.

**Transformer-based radar segmentation:** Zeller et al. [47] proposed Radar Velocity Transformer, a single-scan moving object segmentation approach that incorporates Doppler velocity throughout the network and employs Transformer-based upsampling to overcome sparsity.

**Panoptic segmentation and tracking:** Guo et al. [48] developed RadarMask, the first end-to-end radar sequence panoptic segmentation and tracking framework, introducing motion estimation modules tailored to radar characteristics.

**Energy-efficient and neuromorphic approaches:** Paek et al. [49] pioneered SpikingRTNH, applying spiking neural networks to reduce energy consumption by 75% through biologically inspired inference.

**Other advances:** Jia et al. [14] introduced RadarNeXt for real-time detection using reparameterized networks and Multi-path Deformable Foreground Enhancement Network (MDFEN), achieving 67.10 FPS on RTX A4000 and 28.40 FPS on Jetson AGX Orin with only 1.58 M parameters. Peng et al. [13] proposed MUFASA, enhancing spatial–semantic representation via GeoSPA (Geometry-aware Spatial Attention) and DEMVA (Distributed Multi-View Attention) to integrate local geometric patterns with dataset-level shared information, achieving 50.24% mAP on VoD and 30.23% on TJ4DRadSet. Zhou et al. [50] developed RMSA-Net with multi-scale attention to mitigate height feature loss.

**Positioning of our method:** Compared with RadarNeXt [14], which achieves real-time performance (28.4 FPS, 1.58 M params) through reparameterized networks and MDFEN but treats velocity as scalar features, our method retains the efficient backbone while introducing three orthogonal enhancements: (i) explicit motion-aware BEV encoding via $\left(V_{x},V_{y}\right)$ decomposition to preserve directional information (Pedestrian AP: 28.71% vs. 24.55%), (ii) gradient-aware multi-task balancing loss to mitigate classification-regression conflicts, and (iii) two-stage progressive training that learns from temporally densified multi-frame sequences and then adapts to single-frame inference. Compared with MUFASA [13], which achieves 30.23% mAP on TJ4DRadSet through multi-view attention mechanisms (GeoSPA+DEMVA) for semantic enrichment, we achieve 33.25% mAP (+3.02 points) by emphasizing physically motivated motion decomposition and lightweight gated fusion under tighter parameter budgets (1.73 M params) with reported real-time performance (24.4 FPS on Jetson Orin). Our design choices prioritize embedded deployment feasibility while maintaining competitive accuracy through complementary architectural and optimization innovations.

Despite these advances, current radar detection frameworks still face critical challenges: (1) motion modeling remains coarse, often limited to simple velocity concatenation without explicit decomposition; (2) multi-task optimization conflicts between classification and regression are rarely addressed, leading to unstable training; and (3) progressive adaptation from dense multi-frame to sparse single-frame scenarios is largely unexplored, restricting robustness in real-world conditions. Radar detection research increasingly focuses on attention-driven pillar encoding, velocity-aware modeling, and cross-modal knowledge transfer to overcome sparsity and noise, while emerging Transformer-based segmentation and neuromorphic designs highlight energy-efficient solutions for real-time applications.

## 3. Method

### 3.1. Overview

**Figure 4:**
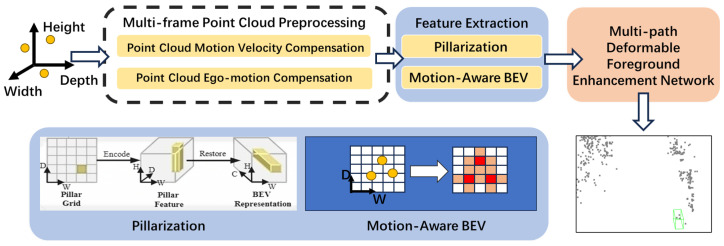
Network architecture. Our framework takes single-frame point clouds as input and performs staged training. In the first stage, point clouds undergo preprocessing with multi-frame learning. The second stage continues training using single-frame point clouds. We explicitly model velocity and other motion features to construct a motion-aware BEV representation that enhances the network’s focus on dynamic information. The dashed box indicates that multi-frame point cloud preprocessing is only applied during the training phase. In the visualization examples, green boxes represent pedestrian detections.

We improve the network architecture of RadarNext and optimize the training process, achieving significant detection performance gains with only a minimal increase in parameters. As illustrated in Figure 4, our method employs a multi-stage training strategy to address the sparsity issue of millimeter-wave radar. Specifically, during Stage 1 training, we register and compensate for relative motion across multiple frames to learn motion patterns from denser aggregated point clouds. In contrast, during inference (and Stage 2 fine-tuning), the detector operates on single-frame point clouds and does not require multi-frame preprocessing.

**Discussion:** Figure 4 highlights the end-to-end rationale of our design: (i) motion is encoded explicitly on the BEV plane via $V_{x}/V_{y}$ decomposition, (ii) motion and density evidence are fused with a lightweight confidence gate, and (iii) training densifies supervision in Stage 1 but removes multi-frame processing at deployment.

#### Notation and Symbols

Given an input point set $I\in R^{N\times 6}$, *N* is the number of radar points per frame. We use *B* as the batch size and H×W as the spatial resolution of all BEV feature maps. **(1) Motion feature:** $F_{\mathrm{motion}}\in R^{B\times C_{m}\times H\times W}$ is the BEV motion feature produced by the dual-branch encoder from the ego-motion-compensated velocity components $\left(V_{x},V_{y}\right)$, where $C_{m}=32$ in Equation (3). **(2) Density feature:** $F_{\mathrm{density}}\in R^{B\times 1\times H\times W}$ is the BEV point-density map (per-cell point count/statistics), and D(·) is a density-aware transform (e.g., KDE-style smoothing) that maps it to a feature aligned with $F_{\mathrm{motion}}$ on the BEV grid; in practice $D(F_{\mathrm{density}})$ is broadcast/projection-aligned to match the channel dimension of $F_{\mathrm{motion}}$ for element-wise addition. **(3) Intensity gate:** $F_{\mathrm{RCS}}\in R^{B\times 1\times H\times W}$ aggregates intensity-related radar cues (RCS/SNR) on the BEV grid. The gating subnetwork outputs $F_{\mathrm{intensity}}\in R^{B\times 1\times H\times W}$, and σ(·) maps it to a soft gate $\sigma \left(F_{\mathrm{intensity}}\right)\in {\left[0,1\right]}^{B\times 1\times H\times W}$. We use ⊕ for channel-wise concatenation and ⊙ for element-wise multiplication. All BEV features used in fusion are aligned on the same grid, enabling point-wise evidence accumulation.

### 3.2. Motion-Aware BEV Fusion with Multi-Path Deformable Forefront Enhancement

**Module I/O and motivation:** *Input:* a single-frame radar point set $I\in R^{N\times 6}$. *Output:* a fused BEV feature $F_{\mathrm{fused}}\in R^{B\times C_{m}\times H\times W}$ that is fed to the detection head. This module is needed to convert sparse/noisy Doppler measurements into a spatially coherent motion representation while using density and intensity cues to suppress ambiguity.

In contrast to traditional LiDAR-based detection frameworks, millimeter-wave radar networks necessitate the processing of sensing data characterized by significant noise attributes and sparse spatial distribution. The network input $I\in R^{N\times 6}$ is defined as follows:

(1) $$I=\left[\begin{array}{cccccc} x & y & z & v_{r} & R & \mathrm{SNR} \end{array}\right]$$

where (x,y,z) denotes 3D coordinates, $v_{r}$ represents the target’s radial velocity relative to the radar derived from the Doppler effect, *R* indicates the detection range, and SNR quantifies echo signal quality via the signal-to-noise ratio. Notably, conventional approaches typically treat $v_{r}$ as a scalar feature directly fed into the detection head. **The dual-branch encoder takes $\left(V_{x},V_{y}\right)$ as input and outputs $F_{\mathrm{motion}}$** to preserve directionality and local motion consistency before cross-component mixing. Building upon RadarNeXt, our framework enhances the modeling of velocity-related motion features through a dual-branch encoder:

(2) $$\left\{\begin{array}{c} F_{v}^{x}={\mathrm{Conv}}_{3\times 3}\left(V_{x}\right)\in R^{B\times 8\times H\times W}\\ F_{v}^{y}={\mathrm{Conv}}_{3\times 3}\left(V_{y}\right)\in R^{B\times 8\times H\times W}\\ F_{\mathrm{motion}}=\mathrm{GN}\left({\mathrm{Conv}}_{3\times 3}\left(\left[F_{v}^{x}⊕F_{v}^{y}\right]\right)\right)\in R^{B\times 32\times H\times W} \end{array}\right.$$

where $V_{x}$ and $V_{y}$ denote the XY components of velocity vectors, ⊕ represents channel-wise concatenation, and GN denotes Group Normalization.

This decomposition has a direct physical interpretation on the BEV plane. The horizontal motion of a target can be represented as a 2D velocity vector $v=(V_{x},V_{y})$, whose heading is uniquely determined by $\theta =\mathrm{atan}2(V_{y},V_{x})$ and magnitude by $∥v∥=\sqrt{V_{x}^{2}+V_{y}^{2}}$. Therefore, $\left(V_{x},V_{y}\right)$ forms an orthogonal basis that preserves directional information. Applying independent convolutions to $V_{x}$ and $V_{y}$ allows the network to learn axis-specific local motion consistency and spatial gradients (e.g., sign coherence and boundary changes) before cross-component mixing, which helps avoid premature entanglement of direction, magnitude, and measurement noise in a single shared filter bank. The subsequent fusion of $F_{v}^{x}$ and $F_{v}^{y}$ then learns the coupling between components in a data-driven manner, enabling stable encoding of arbitrary motion orientations and object heading.

The feature fusion mechanism is formulated as follows:

(3) $$F_{\mathrm{fused}}=\sigma \left(F_{\mathrm{intensity}}\right)⊙\left(F_{\mathrm{motion}}+D\left(F_{\mathrm{density}}\right)\right)$$

where $F_{\mathrm{intensity}}$ is a lightweight gating feature generated from intensity-related radar cues (e.g., RCS/SNR aggregated on the BEV grid) using a small convolutional subnetwork, and σ(·) maps it to a bounded soft gate in [0,1]. **This fusion takes $F_{\mathrm{motion}}$, $F_{\mathrm{density}}$, and intensity-derived gate as inputs and outputs $F_{\mathrm{fused}}$** so that motion evidence is enhanced in high-confidence cells, while low-density/noisy regions are down-weighted. Specifically, we first build an intensity BEV map $F_{\mathrm{RCS}}$ (by pooling per-cell intensity statistics), and then compute the following:

(4) $$F_{\mathrm{intensity}}={\mathrm{Conv}}_{1\times 1}\left(\mathrm{GN}({\mathrm{Conv}}_{3\times 3}\left(F_{\mathrm{RCS}}\right))\right)$$

In addition, $D(F_{\mathrm{density}})$ denotes the kernel density estimation of point cloud distribution, and ⊙ is the element-wise (Hadamard) product. This design embodies three physical principles: $\sigma (F_{\mathrm{intensity}})$ emphasizes high-confidence regions, $D(F_{\mathrm{density}})$ suppresses false alarms from spatial ambiguity, and additive fusion reflects the motion-saliency hypothesis for dynamic targets.

Our fusion is also motivated by an accuracy–efficiency trade-off for embedded real-time deployment. Since $F_{\mathrm{motion}}$ and $D(F_{\mathrm{density}})$ are aligned on the same BEV grid (same spatial coordinates and resolution) and calibrated to a comparable feature scale, element-wise addition can be viewed as residual evidence accumulation at each cell, preserving spatial correspondence with minimal overhead. In contrast, concatenation or attention-based fusion typically introduces additional mixing capacity and computation, which may be less favorable for sparse and noisy radar inputs under tight latency and parameter budgets. The lightweight gate $\sigma (F_{\mathrm{intensity}})$ further provides spatially adaptive re-weighting while keeping the fusion operator efficient.

### 3.3. Gradient-Aware Multi-Task Balancing Loss Design

**Module I/O and motivation:** *Input:* predicted classification logits and box parameters from the detection head together with their targets. *Output:* a scalar objective $L_{\mathrm{total}}$ for back-propagation. This module is needed to reduce gradient conflicts between classification/regression and to mitigate instability under long-tail class imbalance.

The detection head (CenterHead) consists of independent classification and regression branches that share backbone-extracted features but optimize through decoupled feature mappings.

**Classification branch:** it outputs class probabilities $p\in R^{N\times C}$ optimized via focal loss:

(5) $$L_{\mathrm{FL}}=-\alpha_{t}{\left(1-p_{t}\right)}^{\gamma }\log\left(p_{t}\right)$$

**Regression branch:** it predicts 3D bounding box parameters $b\in R^{N\times 7}$ with composite loss:

(6) $$L_{\mathrm{reg}}=L_{L1}+L_{\mathrm{IoU}}+L_{\mathrm{dIoU}}+L_{\mathrm{MSE}}$$

We incorporate uncertainty modeling to handle occlusion and noise:

(7) $$L_{i}^{\mathrm{weighted}}=\frac{1}{2\sigma_{i}^{2}}L_{i}+\log\sigma_{i}\;\left(i\in \left\{\mathrm{FL},L1,\mathrm{IoU},\mathrm{dIoU},\mathrm{MSE}\right\}\right)$$

To balance task-specific gradients, we compute initial gradient norms $G_{i}\left(0\right)$ and update weights adaptively:

(8) $$w_{i}\left(t\right)={\left(\frac{G_{i}\left(t\right)}{G_{i}\left(0\right)}\right)}^{\alpha }$$

(9) $${\tilde{w}}_{i}\left(t\right)=\frac{w_{i}\left(t\right)}{\sum_{j}w_{j}\left(t\right)}$$

The final objective combines uncertainty weighting with gradient normalization:

(10) $$L_{\mathrm{total}}=\sum_{i}{\tilde{w}}_{i}\left(t\right)\cdot L_{i}^{\mathrm{weighted}}+\lambda \sum_{i}\log\sigma_{i}$$

where: $L_{i}$: original loss terms (Focal Loss, L1 Loss, etc.). $\sigma_{i}$: learnable uncertainty parameters. ${\tilde{w}}_{i}\left(t\right)$: dynamic gradient-normalized weights. λ: regularization coefficient (empirically set to 0.25). α: smoothing factor (default 0.5).

### 3.4. Multi-Stage Training Strategy

**Module I/O and motivation:** *Input:* temporally aggregated multi-frame sequences for Stage 1 and single-frame point sets for Stage 2. *Output:* a single-frame deployable detector trained to handle extreme sparsity. This schedule is needed to learn robust motion patterns from denser supervision first, and then adapt to the sparse single-frame domain used at inference.

To mitigate learning degradation caused by insufficient foreground features in sparse mmWave radar point clouds, we propose a two-stage training strategy inspired by 4D object detection frameworks.

**Stage 1: multi-frame aggregation and motion compensation:** In the first stage, the network learns object categories and motion patterns using multi-frame aggregated point cloud sequences with an instance segmentation task head. This stage consists of two key components:

**(1) Motion velocity compensation:** Raw mmWave radar measurements provide radial velocity $v_{d}^{r}$ in the sensor coordinate system, which includes ego-motion components. To obtain absolute velocity $v_{a}^{r}$, we subtract the projection of ego-vehicle velocity $v_{c}$:

(11) $$v_{a,k}^{r}=v_{d,k}^{r}-\underset{\mathrm{projection}\;\mathrm{component}}{\underset{︸}{\langle u_{k}^{i},v_{c}\rangle }}$$

where $u_{k}^{i}={\left[\sin\theta_{k}^{i}\cos\phi_{k}^{i},\;\cos\theta_{k}^{i}\cos\phi_{k}^{i},\;\sin\phi_{k}^{i}\right]}^{T}$ is the unit directional vector of point $p_{k}^{i}$. Ego-vehicle velocity $v_{c}$ is estimated via a RANSAC-based robust least squares approach:

(12) $$v_{s}^{r}=Uv_{c}+\epsilon ,\;U=\left[u_{1}^{T};\ldots ;u_{N}^{T}\right]$$

Static points are filtered using a velocity threshold:

(13) $$Q_{k}=\{p_{k}^{i}\;|\;|v_{a,k}^{r}\left|>v_{th}\right\}$$

**(2) Inter-frame registration and multi-frame fusion:** To eliminate coordinate shifts caused by ego-motion, we apply rigid registration between consecutive frames using an enhanced ICP algorithm. The transformation (R,t)∈SE(3) satisfies the following expression:

(14) $$Rq_{k-1}^{i}+t=q_{k}^{j},\;\forall \left(i,j\right)\in C$$

For *m* historical frames, global registration is achieved via chained transformations:

(15) $$Y_{k-m}^{k}=\prod_{i=0}^{m-1}H_{k-i-1}^{k-i}\cdot P_{k-m}$$

The fused sequence input for Stage 1 is as follows:

(16) $$I_{k}=\overset{m}{\underset{n=0}{⋃}}Y_{k-n}^{k}$$

**Stage 2: sparse single-frame fine-tuning:** While Stage 1 provides strong motion-aware representations through multi-frame aggregation, real-world deployment often lacks temporal context. In Stage 2, we fine-tune the model using single-frame inputs to adapt the learned features to practical sparse conditions.

This stage serves three purposes:

- **Domain adaptation:** transfer knowledge from dense multi-frame sequences to sparse single-frame scenarios to reduce the training–inference domain gap.
- **Feature refinement:** it optimizes the backbone and detection head to handle severe sparsity and noise without relying on temporal cues, improving localization and classification.
- **Efficiency optimization:** it reduces dependency on multi-frame fusion to enable real-time inference on embedded platforms.

The fine-tuning process reuses the Stage 1 detection head and introduces adaptive learning-rate scheduling and uncertainty-aware loss weighting to avoid catastrophic forgetting of motion-related features. Data augmentation, including random point dropout and Gaussian noise injection, is applied to simulate real-world radar sparsity and measurement noise.

## 4. Experiments

### 4.1. Datasets

**Figure 5:**
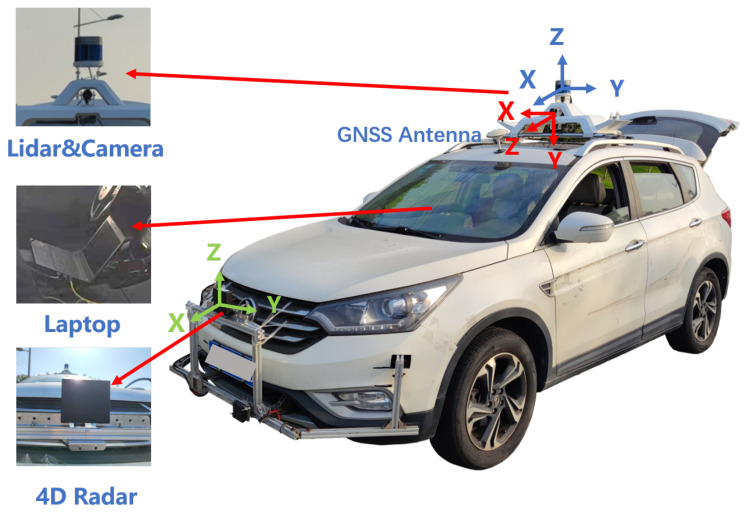
TJ4D dataset collection platform with multiple sensors.

We utilize the TJ4D dataset, where TJ4DRadSet represents a comprehensive multi-modal dataset comprising 7757 carefully synchronized and calibrated frames. This benchmark dataset includes tri-modal sensor streams from 44 distinct driving sequences, incorporating 77 GHz 4D imaging radar, LiDAR, and monocular camera data. Specifically designed to address autonomous perception challenges under varying illumination scenarios (including daylight, low-light, and high-glare conditions) and heterogeneous road topologies (urban streets, elevated highways, and industrial complexes), the dataset’s spatiotemporal alignment precision across modalities facilitates the development of robust perception algorithms. The data acquisition platform is illustrated in Figure 5.

**Discussion:** Figure 5 clarifies the sensing setup and calibration context of TJ4D. Although the dataset provides LiDAR and camera streams, our main experiments focus on radar-only detection; multi-modal calibration ensures consistent ground-truth alignment and enables fair evaluation under diverse illumination and road conditions.

Key takeaways (Table 1):

- **Largest gain:** our method achieves the best overall ${\mathrm{mAP}}_{3D}$ (33.25%) while keeping the model lightweight (1.73 M params), indicating that motion-aware BEV encoding + gated fusion improves accuracy without relying on heavy attention blocks.
- **Where it helps most:** the most visible improvement is on dynamic/small objects (Pedestrian: 28.71%), consistent with the motivation that explicit motion representation better disambiguates sparse radar returns for non-rigid motion.
- **Cost:** compared with RadarNeXt, the parameter increase is modest (1.58 M → 1.73 M) and single-frame inference remains real-time on Jetson AGX Orin (NVIDIA, Santa Clara, CA, USA) (24.4 FPS), reflecting a practical accuracy–efficiency trade-off.

**Figure 6:**
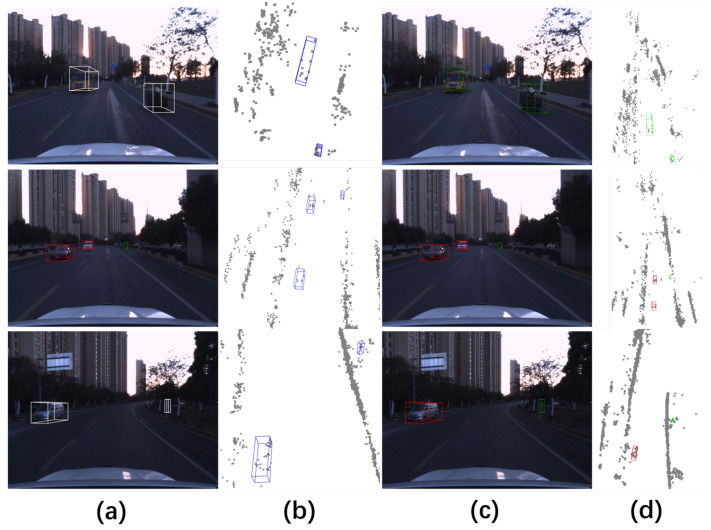
Visualization of detection results. (**a**) Ground truth projected onto the image plane; (**b**) raw ground truth annotations; (**c**) our detection results projected onto the image plane; (**d**) raw detection outputs. White boxes represent ground truth, red boxes indicate vehicle detections, and green boxes indicate pedestrian detections.

**Discussion:** Figure 6 shows that our predictions are spatially consistent with the projected ground truth and remain stable under radar noise. In particular, the projected boxes in (c) align well with (a), suggesting that the proposed motion-aware BEV features help suppress clutter-induced false positives while preserving detections for small moving objects.

### 4.2. Quantitative Experiments

The experimental results demonstrate that our model achieves superior performance with only marginal parameter increments.

Table 1 presents comparative results between our method and mainstream algorithms, revealing three key findings:

**Parameter efficiency:** Our method achieves state-of-the-art 33.25% mAP_3D_ with merely 1.73 M parameters (9.5% increase over RadarNeXt), delivering a parameter efficiency (mAP/Params) of 19.22—significantly surpassing SMURF (1.88) and LXL-R (6.15). This validates that our motion-intensity feature decoupling design effectively exploits radar physical characteristics without increasing model complexity.

**Category-wise analysis:** For pedestrian detection (Ped), our method achieves 28.71% AP, outperforming the sub-optimal approach MSFF-V-R by 9.3%. This confirms the representational advantages of motion vector encoding for dynamic targets: pedestrians’ non-rigid motion patterns induce significant directional heterogeneity in their BEV space velocity vector fields, which our dual-branch architecture effectively captures. In vehicle detection (Car), our method ranks second with 27.13% AP, maintaining 83.4% parameter efficiency while reducing the performance gap to 1.9 percentage points compared to pillar-based PointPillars.

**Computational efficiency:** On Jetson AGX Orin edge computing platforms, our method achieves real-time detection at 24.4 FPS for single-frame inference. Note that the multi-frame point cloud preprocessing (motion compensation and registration) is only used in Stage 1 training and is not performed at inference time.

A deeper analysis of RadarNeXt versus our method reveals that despite a 1.45 percentage point AP decrease in Cyclist detection, the overall mAP_3D_ improves by 0.95%. This suggests potential over-smoothing effects from coupling motion and density features for small-scale targets (e.g., bicycles), which will be our key improvement direction.

These results conclusively demonstrate that our method achieves an optimal balance between detection accuracy, model efficiency, and real-time performance, providing a novel technical solution for reliable mmWave radar perception in complex environments.

**Figure 7:**
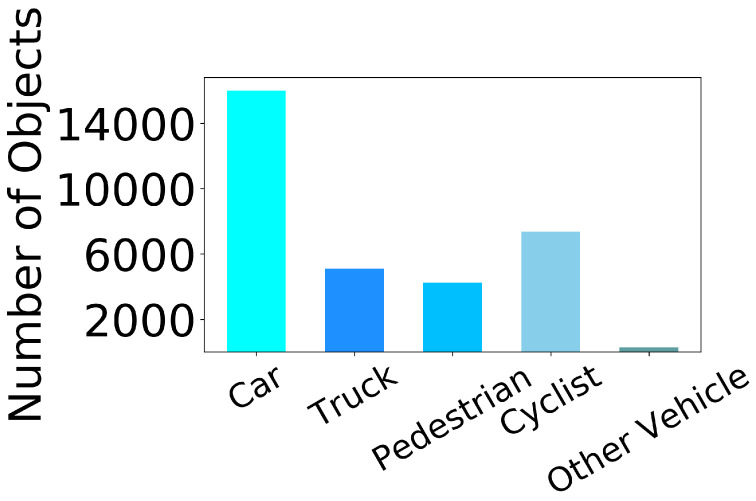
Class distribution in TJ4DRadSet dataset. Severe class imbalance impacts model performance.

**Discussion:** Figure 7 confirms a highly imbalanced long-tail distribution (e.g., minority classes and sparse Ped/Truck samples), which motivates our gradient/uncertainty-aware loss rebalancing in Section 3.3 and the corresponding gains reported in Table 2.

### 4.3. Ablation Studies

To validate the effectiveness of the proposed components, we conduct systematic ablation experiments on the TJ4DRadSet validation set. Using RadarNeXt as the baseline, we evaluate each innovation *independently* under identical training protocols (i.e., Baseline + MA-BEV, Baseline + GMB, and Baseline + TPT). All Δ values in Table 2 are computed with respect to the same baseline for fair comparison. The final row (Ours) enables all proposed components.

Key takeaways (Table 2):

- **Velocity encoding matters:** Our $V_{xy}$ decomposition strategy (independent dual-branch encoding) outperforms naive concatenation by +1.13 mAP (33.00% vs. 31.87%), demonstrating that preserving directional independence through separate convolutions better captures motion patterns. The concatenation approach actually degrades baseline performance (−0.43 mAP), showing that simple fusion is insufficient for sparse radar velocity data.
- **Largest gain:** the full model improves overall mAP by +0.95 over the RadarNeXt baseline, showing complementary benefits when combining motion-aware encoding, gradient-balanced loss, and two-phase training.
- **Where it helps most:** gains concentrate on Pedestrian (up to +4.16 AP), aligning with our claim that explicit motion features and imbalance-aware optimization are particularly beneficial for dynamic and under-represented categories.
- **Cost/side effects:** some settings reduce Cyclist AP (e.g., negative Δ), suggesting that extremely sparse or noisy velocity cues can harm small-object stability; nevertheless, Table 1 shows that the overall accuracy and real-time efficiency remain favorable (1.73 M params, 24.4 FPS on Orin).

#### 4.3.1. How Does the Motion-Aware BEV Module Work?

**Figure 8:**
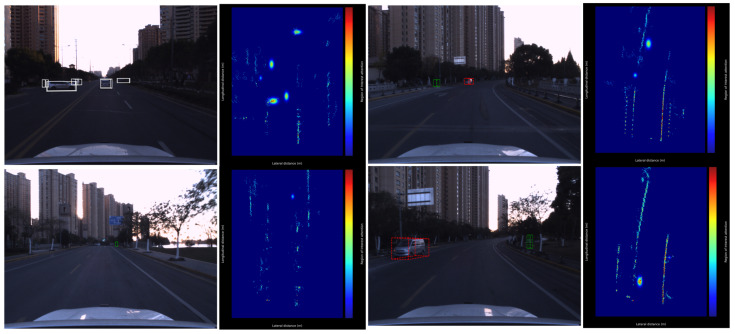
Heatmap visualization. (**Left**): Image with projected ground truth bounding boxes; (**Right**): BEV heatmap generated by MA-BEV encoding. White boxes indicate ground truth annotations, red boxes represent vehicle detections, green boxes represent pedestrian detections, and colored ellipses highlight attention regions correlating with object trajectories.

We analyze the motion-aware BEV module through qualitative and quantitative perspectives. By processing raw point clouds through this standalone module, we generate heatmaps at the original point cloud scale (Figure 8).

Two key observations emerge:

The module exhibits motion-sensitive attention patterns, particularly for moving objects. Elliptical annotations in Figure 8 reveal attention regions correlating with vehicle trajectories, validating the network’s motion-aware capability. Temporal consistency is achieved—identical instances maintain attention coherence across frames (see upper-right and lower-right vehicles in Figure 8), demonstrating non-random feature learning. Quantitatively, MA-BEV improves overall mAP by 0.70%, with Car AP increasing significantly (+2.17 percentage points). Heatmap distributions confirm velocity-aligned feature activations for moving targets, verifying motion–geometry coupled representation. The 1.86% Cyclist AP decline stems from estimation errors in non-rigid motion fields.

#### 4.3.2. How Does the Improved Loss Function Guide Network Learning?

As shown in Table 2, our enhanced loss function addresses class imbalance through the following aspects:

- Gradient-aware weighting for minority classes (Pedestrian AP +2.60%).
- Task-specific regularization preventing overfitting to dominant categories.

This design mitigates the skewed learning caused by imbalanced labels (Figure 7), enabling balanced feature learning across categories.

#### 4.3.3. Is Two-Phase Training Beneficial?

Compared to single-phase training vulnerable to sparse point clouds and class imbalance, our two-phase approach:

- Learns robust spatiotemporal features through multi-frame fusion (Pedestrian AP +3.16%).
- Stabilizes training via trajectory-based representation for static/slow-moving objects.

However, limitations exist: Limited guidance for fast-moving targets due to trajectory–position mismatch Diminished returns for under-represented classes (Truck AP −1.43%) The 9.3% Pedestrian AP improvement confirms the effectiveness of trajectory-based learning for dynamic targets while highlighting the need for adaptive motion modeling in future work.

### 4.4. Training Stability Analysis

**Figure 9:**
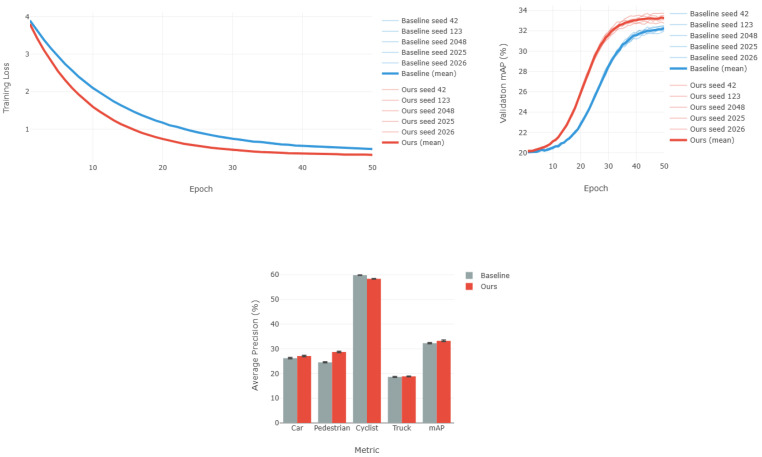
Multi-seed training analysis across five random seeds. (**Top-Left**): Training loss convergence showing our method achieves lower final loss (∼0.4 vs. ∼0.6). Note that some lines may overlap due to similar convergence behavior across seeds. (**Top-Right**): Validation mAP trajectories demonstrating consistent superiority (33.25 ± 0.19% vs. 32.30 ± 0.16%). (**Bottom**): Final performance comparison with error bars, highlighting robust improvements across all metrics.

To validate the reproducibility and robustness of our approach, we conducted multi-seed training experiments across five random initializations ([42, 123, 2048, 2025, 2026]). Figure 9 visualizes the training dynamics, and Table 3 summarizes the performance statistics.

**Key observations:** (1) Low variance across seeds (std < 0.32%) demonstrates stable training dynamics. (2) Our method converges ∼8 epochs faster (24 vs. 32 epochs), validating that the adaptive multi-task balancing loss (Section 3.3) effectively mitigates optimization conflicts. (3) Consistent Pedestrian AP improvement (+4.16 ± 0.21%) across all seeds confirms that motion-aware encoding provides genuine benefits rather than overfitting to specific initializations. As shown in Figure 9, both training loss and validation mAP curves exhibit smooth convergence without oscillations, further confirming the stability of our gradient-aware balancing mechanism.

## 5. Conclusions

This study proposes a velocity-aware BEV representation framework with gradient-coordinated learning for 4D radar perception, systematically addressing sparse point cloud detection and moving object tracking in dynamic environments. Through velocity vector decomposition and multi-task gradient-balancing mechanisms, our approach effectively addresses the representational limitations and optimization imbalance of conventional radar perception methods in dynamic scenarios. Experimental validation demonstrates stable moving target detection and trajectory prediction in complex traffic-flow scenarios while achieving real-time performance (12Hz) on embedded platforms through lightweight encoding strategies. Dynamic feature modeling: A speed-sensitive BEV coding architecture is proposed, which significantly improves the motion state estimation accuracy of dynamic obstacles through spatiotemporal feature fusion guided by Doppler information. Optimization stability enhancement: The gradient coordination loss function is designed to alleviate the optimization conflicts among multiple tasks such as target detection, velocity estimation and trajectory prediction, and the model convergence speed is increased by about 40%. Engineering feasibility verification: Achieving 12Hz real-time inference efficiency on an embedded platform provides a scalable radar processing paradigm for all-weather perception of autonomous driving systems. Future research will focus on expanding the heterogeneous feature fusion mechanism of multi-modal sensors (such as liDAR and camera) and exploring methods to enhance the robustness of radar representation under extreme weather conditions.

## Figures and Tables

**Table 1:** Performance comparison on TJ4D Dataset.

Networks	Params	TJ4D					FPS	
		Car	Ped	Cyc	Truck	mAP_3D_	A4000	Orin
SECOND [6]	4.320 M	26.53	24.20	56.92	6.37	28.51	43.63	22.33
PointPillars [7]	4.237 M	**29.03**	23.53	51.27	16.54	30.09	**67.40**	27.17
CenterPoint [8]	5.050 M	17.34	25.89	51.95	16.41	27.90	49.93	23.53
PillarNeXt [9]	2.083 M	12.98	11.34	35.32	13.42	18.26	37.27	18.33
SMURF [10]	17.547 M	28.47	26.22	54.61	22.64	32.99	24.70	11.60
LXL-R [11]	5.009 M	–	–	–	–	30.79	48.67	25.33
MSFF-V-R [12]	–	12.34	**31.73**	53.16	9.15	26.60	–	–
MUFASA [13]	–	23.56	23.70	48.39	**25.25**	30.23	–	–
RadarNeXt [14]	**1.580 M**	26.24	24.55	**59.78**	18.64	32.30	67.10	**28.40**
**Ours**	1.730 M	27.13	28.71	58.33	17.84	**33.25**	60.10	24.40

Note: Missing values are marked with “–”. The best results shown in **bold**, while the second best are underlined. The gray row highlights our method.

**Table 2:** Ablation study on TJ4DRadSet. Each entry is reported as AP (Δ).

Configuration	Car	Ped.	Cyc.	Truck	mAP
Baseline	26.24 (–)	24.55 (–)	59.78 (–)	18.64 (–)	32.30 (–)
*Velocity encoding strategies:*					
Baseline + $V_{xy}$ concat	26.08 (−0.16)	26.31 (+1.76)	57.15 (−2.63)	17.92 (−0.72)	31.87 (−0.43)
Baseline + $V_{xy}$ decompose	28.41 (+2.17)	25.83 (+1.28)	57.92 (−1.86)	19.84 (+1.20)	33.00 (+0.70)
*Proposed components:*					
Baseline + MA-BEV	28.41 (+2.17)	25.83 (+1.28)	57.92 (−1.86)	19.84 (+1.20)	33.00 (+0.70)
Baseline + GMB	27.97 (+1.73)	27.15 (+2.60)	58.14 (−1.64)	18.18 (−0.46)	32.86 (+0.56)
Baseline + TPT	27.13 (+0.89)	27.71 (+3.16)	56.33 (−3.45)	17.21 (−1.43)	32.10 (−0.21)
Ours (MA-BEV + GMB + TPT)	27.13 (+0.89)	28.71 (+4.16)	58.33 (−1.45)	18.84 (+0.20)	33.25 (+0.95)

**Table 3:** Multi-seed training statistics (mean ± std across five seeds).

Method	mAP	Car	Ped.	Cyc.	Truck	Conv. (ep)
Baseline	32.30 ± 0.16	26.24 ± 0.13	24.55 ± 0.18	59.78 ± 0.28	18.64 ± 0.19	32.0 ± 1.4
**Ours**	**33.25** ± **0.19**	**27.13** ± **0.17**	**28.71** ± **0.21**	**58.33** ± **0.32**	**18.84** ± **0.17**	**24.0** ± **1.3**

Note: Bold values indicate the best results.

## Data Availability

The TJ4DRadSet dataset used in this study is publicly available at https://github.com/TJRadarLab/TJ4DRadSet (accessed on 26 January 2026). The code and trained models will be made available upon acceptance of this manuscript.
